# Association between physical activity and executive function of Chinese adolescents aged 13–18

**DOI:** 10.3389/fped.2025.1576546

**Published:** 2025-07-15

**Authors:** Yuan Liu, Xiaojian Yin, Yi Sun, Feng Zhang, Cunjian Bi, Yaru Guo, Pengwei Sun, Hong Jun, Yanyan Hu, He Liu

**Affiliations:** ^1^ Physical Education College of Shanghai University, Shanghai, China; ^2^ Department of Physical Education, Shanghai Institute of Technology, Shanghai, China; ^3^ College of Physical Education, Ludong University, Yantai, Shandong, China; ^4^ Key Laboratory of Adolescent Health Assessment and Exercise Intervention of the Ministry of Education, East China Normal University, Shanghai, China; ^5^ College of Physical Education and Health, East China Normal University, Shanghai, China; ^6^ Sports Health Promotion Center, Chizhou University, Chizhou, China

**Keywords:** inhibitory control, working memory, cognitive flexibility, physical activity, executive function, adolescents

## Abstract

**Objective:**

This study aimed to investigate the relationship between physical activity (PA) and executive function (EF) among adolescents in China.

**Methods:**

Using a stratified cluster random sampling, we recruited 4,991 adolescents from 11 Chinese cities, including Urumqi, Lhasa, and Naqu etc. Participants completed execution function test and PA questionnaire survey. Data were analyzed using one-way ANOVA, *Pearson* correlation analysis, and 30-minute isotemporal substitution models (ISM) to assess the association between PA and EF.

**Results:**

Daily moderate-to-vigorous physical activity (MVPA) time in both boys and girls was negatively correlated with inhibitory control RTs (*r* = −0.279, −0.173, *P* < 0.01), 2back-RTs (*r* = −0.367, −0.268, *P* < 0.01), and cognitive flexibility RTs (*r* = −0.283, −0.305, *P* < 0.01). Replacing 30 min of sedentary behaviour (SB) with MVPA was significantly shorter RTs in inhibitory control (*P* < 0.05), 2back tasks (*P* < 0.01), and cognitive flexibility (*P* < 0.01). Similarly, substituting 30-min of light physical activity (LPA) with MVPA led to reduced RTs in all three EF domains (*P* < 0.05 for inhibitory control; *P* < 0.01 for others). After adjusting for covariates, a U-shaped dose-response relationship emerged between MVPA duration and EF performance. Adolescents with 59.02–60.88 min/day of MVPA (the relatively high-level group) demonstrated the lowest RTs in inhibitory control, working memory, and cognitive flexibility (*P* < 0.05).

**Conclusions:**

After controlling for confounders, MVPA exhibited an inverted U-shaped relationship with inhibitory control, working memory, and cognitive flexibility. Optimal EF improvement was observed at 59.02–60.88 min/day of MVPA, suggesting this range may be most beneficial for adolescents’ executive function.

## 1 Introduction

Executive function (EF) has become an important area of research in various academic fields including psychology, education, and physical education. With the increase of interdisciplinary research, more scholars have focused on the relationship between physical activity (PA) and EF of adolescents. Previous studies have indicated that PA is most closely associated with the sub-functions of EF (inhibitory control, working memory, and cognitive flexibility). (1). However, research on the correlation between PA and EF in adolescents remains inconclusive, Some studies have found a significant association between PA and EF among adolescents (2, 3) and long-term regular PA has been shown to benefit the improvement of EF of adolescents (1, 4). In addition, numerous studies suggest that regularly increasing PA in adolescents’enhances both EF and learning ability (5, 6), while a lack of long-term PA has negative effects on EF (7).

However, others studies havefound no significant difference between PA and EF in adolescents (8–10). These discrepancies may be due to factors such as differenences in sample sizes and selection of subjects, variations in methods for assessing PA and EF, and the control of independent variables. Additionally, research on the relationship between different levels of sedentary behavior (SB), PA (including LPA, low physical activity; MVPA, moderate-to-vigorous physical activity; TPA, total physical activity.) and the sub-functions of EF among adolescents remains limited and lacks depth.

Building on previous studies, PA is recognized as an important, controllable, and easily modifiable factor affecting the EF of adolescents. Therefore, this study aims to investigate the relationship between PA and EF in adolescents, with a particular focus on the optimal amount of daily PA required to achieve the greatest improvement in EF. The findings of the study will help identify effective strategies to promote the healthy development of EF among adolescents.

## 2 Methods

### 2.1 Data sources and participants recruitment

**Figure 1:**
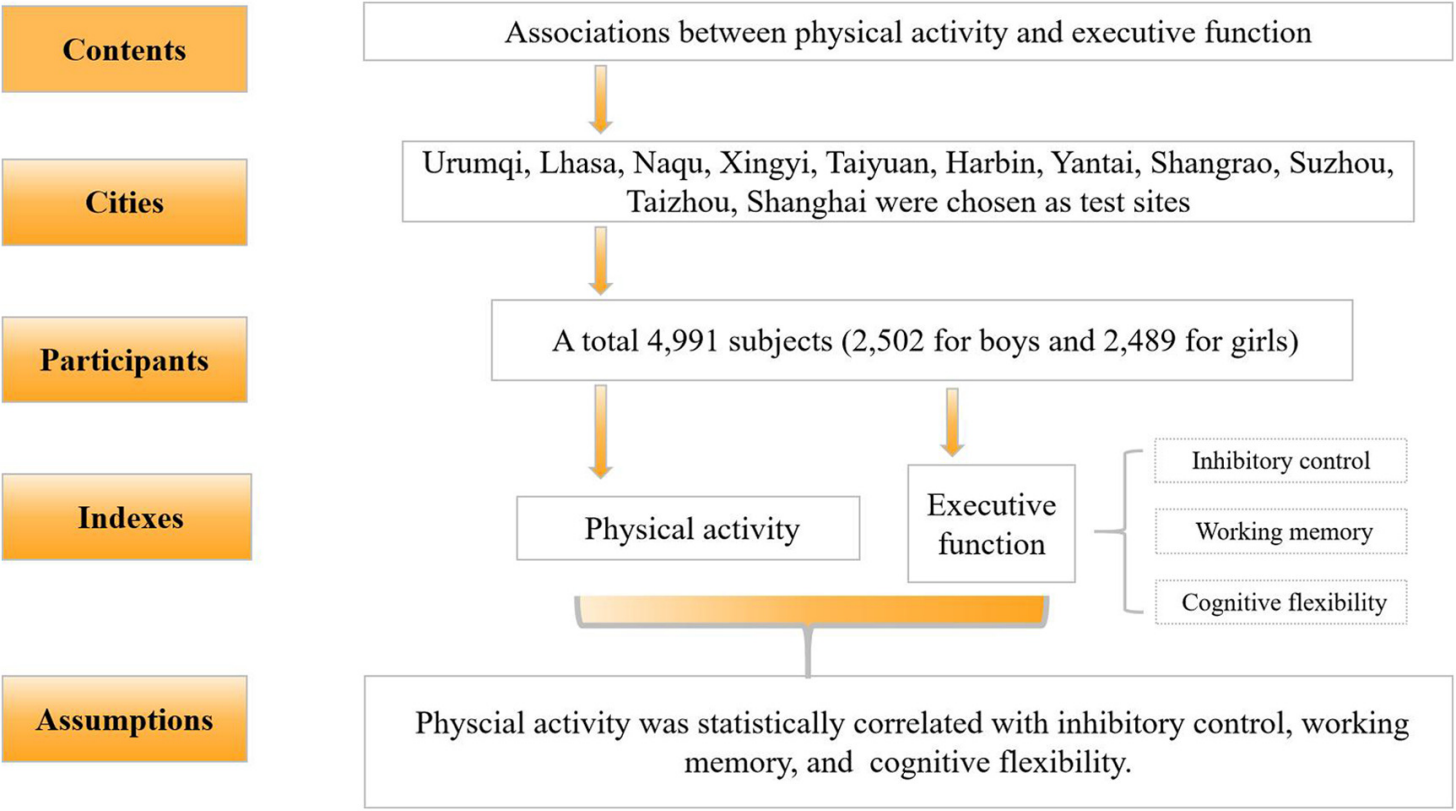
Flowchart of the study design.

A stratified random cluster sampling method was adopted to select test sites based on surveys of the physical health of Chinese students. Eleven cities —Urumqi, Lhasa, Naqu, Xingyi, Taiyuan, Harbin, Yantai, Shangrao, Suzhou, Taizhou, Shanghai —were included as test sites in this study. To minimize confounding factors, the participants with locomotor deficits or neurodevelopmental disorders that could potentially affect executive function (EF) were excluded prior to E*F* testing. Additionally, after conducting the E*F* test, only data from participants who achieved an accuracy rate exceeding 80% in each task were retained as valid. The inclusion criteria for participants were as follows: (1) aged 13–18 years, (2) no color blindness, and (3) no serious physical or mental illness. Following the exclusion of invalid data and outliers, and ensuring a gender ratio of approximately 1:1, a total of 4,991 participants (2,502 boys, 50.95%; 2,489 girls, 49.05%) were included in the final analysis. Data collection spanned from August 2023 to December 2024 across China (Figure 1).

### 2.2 Physical activity

To assess physical activity (PA) levels, we adopted the Health Industry Standard of the People's Republic of China (Physical Activity Level Evaluation for Children and Adolescents Aged 7–18 Years) as the reference framework (11). The questionnaire demonstrated good internal consistency (Cronbach's *α* = 0.717, *P* < 0.01) and high structural validity (12). Additionally, its test-retest reliability (*r* = 0.69) exceeded the minimum threshold of 0.60 (13), further supporting its robustness. Exercise intensity, as a critical dimension of physical activity, was quantified in Metabolic Equivalent of Task (MET) units (14). The World Health Organization (WHO) defines it as “the rate of work performed or the magnitude of force exerted during a given exercise or physical activity” (15). In accordance with the Ainsworth et al. (16) classification system for physical activity intensity, MET-based thresholds delineate three distinct categories:

Sedentary behavior (SB): <1.5 METs;
Low-intensity physical activity (LPA): ≥1.5 METs but <3 METs;
Moderate-intensity physical activity (MPA): ≥3 METs but ≤6 METs;
Vigorous-intensity physical activity (VPA): >6 METs.

### 2.3 Executive function and related assessments

The EF task-cuing paradigm was developed by Aiguo et al. (17) and includes tests for inhibition (Eriksen flanker task) (18), working memory test by (N-back task) (19), and cognitive flexibility (more-odd shifting task) (20). Reaction time (RT) served as the primary outcome measure for all tasks, with shorter RTs indicating superior performance. The difference in RT between incongruent and congruent trials was used to estimate inhibition. The 1-back and 2-back tests were used to assess working memory. The difference in RT between heterogeneous and homogeneous trials was used to estimate cognitive flexibility. The E*F* tests were conducted using a program created by the E-prime 1.1 software system (Psychology Software Tools Inc., Pittsburgh, USA).

### 2.4 Isotemporal substitution models

The 30-min ISM was employed to evaluate the relationship between between the mutual substitution of SB, LPA, and MVPA and sub-function of EF. Prior to model execution, all activity durations (SB, light LPA, and MVPA) were normalized by dividing by 30-minute time units, consistent with physical activity guideline recommendations (21). Here, an increase of 1 unit represents an additional 30 min per day. Constructing the isotemporal substitution model involves three steps: building single-factor models, developing the partition model, and performing isotemporal substitution. The single-factor model examines the relationship between each activity behavior and the dependent variable, adjusted for confounders but without accounting for other activity types or considering substitution effects. Taking SB as an example—the single-factor model is: dependent variable = (*β*_1_) SB + (*β*_5_) covariates. The partition model is: dependent variable = (*β*_1_) SB + (*β*_2_) LPA + (*β*_3_) MVPA + (*β*_5_) covariates, where the coefficients of activity behaviors indicate the improvement of time changes while keeping other activities constant. The isotemporal substitution model represents the improvement of replacing one activity type with another while keeping the total wear time constant. For example, replacing SB with LPA or MVPA, the isotemporal substitution model is: dependent variable = (*β*_2_) LPA + (*β*_3_) MVPA + (*β*_4_) total activity time + (*β*_5_) covariates.

### 2.5 Covariates

The study covariates included age (22), gender (23), site location (24), socioeconomic status (25), body mass index (26), waist circumference (27), breakfast frequency per week (28), and sugar-sweetened beverage (29) consumption. Data on age, gender, site location, breakfast frequency, and SSB consumption were collected using questionnaires derived from the Chinese National Survey on Students’ Constitution and Health (CNSSCH) (30). Information on breakfast intake was obtained by separate questionnaires with four questions in the past 7days. Respectively are “How many times did you have breakfast? (≤2, 3–4, 5–7)”, “What is the frequency of eating milk? (≤2, 3–4, 5–7)”, “How many times did you drink sugar-sweetened beverage (SSB)? (≤2, 3–4, 5–7)”, and “What is the frequency of eating soybean product consumption in the past 7 days? (≤2, 3–4, 5–7)”. Physical health, height, weight, and WC of the participants were taken according to the implementation rules of the 2014 national survey (31). BMI was calculated as weight (kg) divided by height (m) square.

### 2.6 Statistical analyses

Firstly, the normality test was performed on the data. If the data followed a normal distribution, mean ± standard deviation (SD) was used to describe the data. For data that did not follow a normal distribution, quartiles (<*P*_25_, *P*_25_–*P*_50_, *P*_50_–*P*_75_, and >*P*_75_) were used to describe the data (32). According to MVPA, the cut-off points were based on *P*_25_, *P*_50_, and *P*_75_ percentiles for each age and gender (33, 34) categorizing participants into the following MVPA groups: lowest MVPA group (MVPA < *P*_25_), lower MVPA group (*P*_25_ ≤ MVPA < *P*_50_), higher MVPA group (*P*_50_ ≤ MVPA ≤ *P*_75_), and highest MVPA group (MVPA > *P*_75_). The correlation between PA and RT of EF was assessed by *Pearson* correlation. A linear regression model was applied to diagnose multicollinearity, aiming to minimize distortion caused by high correlations between independent variables. After addressing multicollinearity of the independent variables, a linear regression model was applied to explore the effects of SB, LPA, MVPA and TPA on the EF of adolescents. Corresponding data analysis was conducted using SPSS 26.0 software, with *α* = 0.05 set as the two-tailed test level. Image processing was performed using Origin Pro9.1 software.

## 3 Results

### 3.1 Descriptive characteristics for various variables

The study included 2,950 participants (59.11%) from urban areas and 2041 (40.89%) from rural areas (Table 1). Socialeconomic status (SES), distribution was as follows: high SES (*n* = 968, 19.40%), middle SES (*n* = 2,071, 41.49%), and low SES (*n* = 1,952, 39.11%), with the middle SES group representing the largest proportion. Participant characteristics stratified by sedentary behavior (SB), light physical activity (LPA), moderate-to-vigorous physical activity (MVPA), and total physical activity (TPA) levels are detailed in Table 1.

**Table 1:** Characteristics of the participants.

Characteristics	Overall (*N* = 4,991)
Age {y [mean (SD)]}	15.4 (1.7)
Sex [*n* (%)]	
Boys	2,502 (50.95)
Girls	2,409 (49.05)
Residential district, *n* (%)	
Urban	2,950 (59.11)
Rural	2,041 (40.89)
SES, n(%)	
Low SES	968 (19.39)
Middle SES	2,071 (41.49)
High SES	1,952 (39.12)
The frequency of eating breakfast, *n* (%)	
5–7 times/week	2,470 (49.49)
3–4 times/week	1,389 (27.83)
≤2 times/week	1,132 (22.68)
The frequency of eating milk, *n* (%)	
5–7 times/week	2,194 (43.96)
3–4 times/week	1,118 (22.40)
≤2 times/week	1,679 (33.64)
The frequency of eating SSB, *n* (%)	
5–7 times/week	908 (18.19)
3–4 times/week	1,191 (23.86)
≤2 times/week	2,892 (57.95)
The frequency of eating soybean product consumption, *n* (%)	
5–7 times/week	1,405 (28.15)
3–4 times/week	1,322 (26.49)
≤2 times/week	2,264 (45.36)
BMI, *n* (%)	
Underweight	384 (7.69)
Normal	3,516 (70.45)
Overweight	687 (13.76)
Obesity	404 (8.10)
WC (Mean, SD)	70.96 (10.01)
CRF (Mean, SD)	49.08 (9.33)
Inhibitory control RTs (Mean, SD)	8.44 (9.47)
Working memory RTs (Mean, SD)	1,060.72 (302.58)
Cognitive flexibility RTs (Mean, SD)	305.62 (171.64)
SB (Mean, SD)	671.93 (174.88)
LPA (Mean, SD)	33.68 (19.70)
MVPA (Mean, SD)	43.42 (26.53)
TPA (Mean, SD)	77.02 (22.17)

SES, social economic status; SSB, sugar-sweetened beverage; BMI, body mass index; WC, waist circumference; CRF, cardiorespiratory fitness; RT, reaction time; SB, sedentary behavior; LPA, low physical activity; MVPA, moderate-to-vigorous physical activity; TPA, total physical activity.

### 3.2 Pearson correlation of SB, LPA, MVPA, and TPA in different EF RTs groups

**Figure 2:**
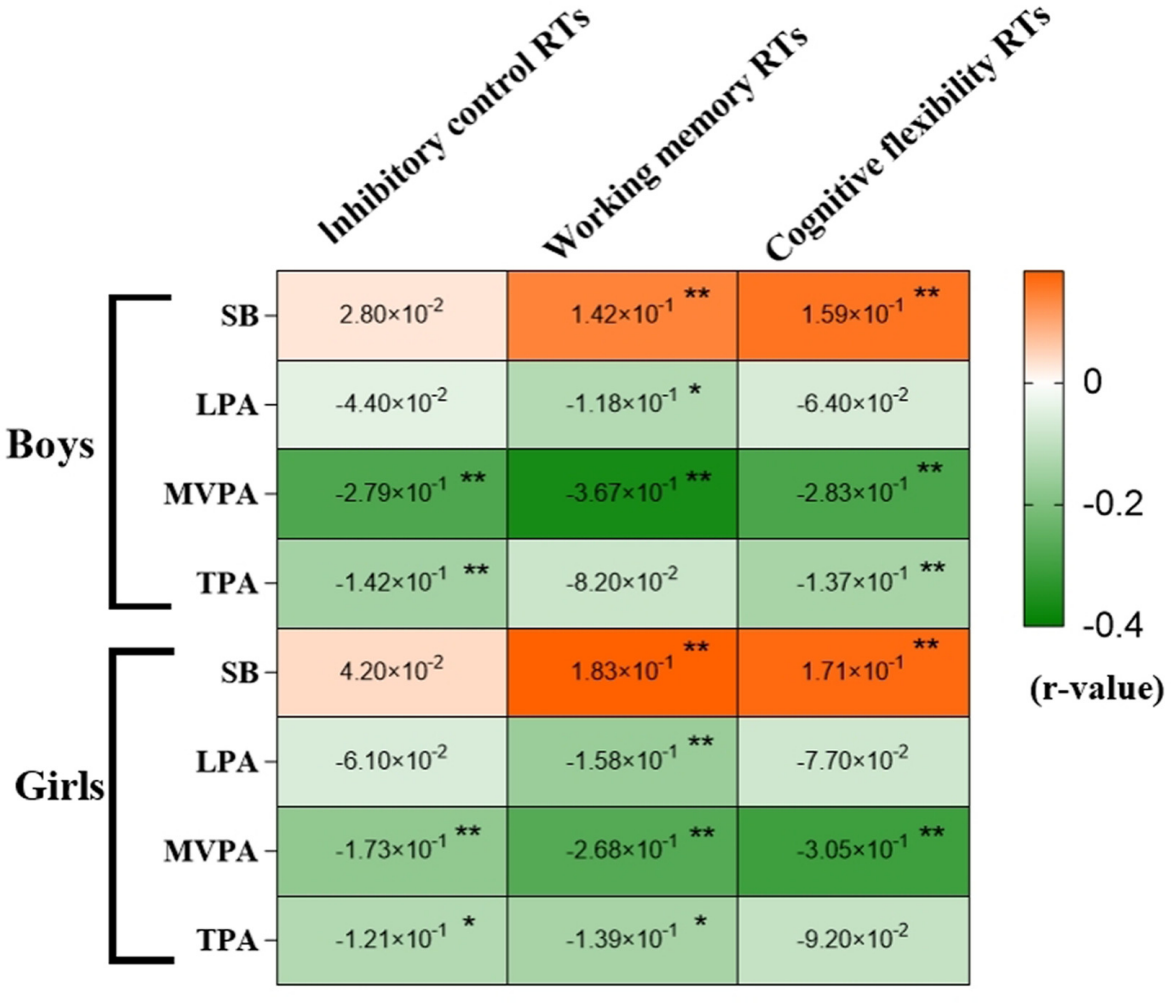
Heat map of correlation between SB, LPA, MVPA, TPA, and EF RTs. RT, reaction time; SB, sedentary behavior; LPA, low physical activity; MVPA, moderate-to-vigorous physical activity; TPA, total physical activity; *P < 0.05, **P < 0.01.

Figure 2 presents the Pearson correlation coefficients between SB, LPA, MVPA, and TPA with RTs across different executive function domains, stratified by gender. Positive correlations are denoted in orange, while negative correlations are indicated in green, with color intensity reflecting the strength of association. There was no significant correlation between daily SB and inhibitory function RTs in the youth cohort. However, daily MVPA time exhibited a significant negative correlation with inhibitory function RTs (*P* < 0.05). Daily SB time showed a positive correlation with working memory RTs in both males and females (*r* = 0.142, 0.183, *P* < 0.01). Conversely, daily LPA and MVPA time were negatively correlated with working memory RTs in both genders (*P* < 0.05). A positive correlation was observed between daily SB time and cognitive flexibility RTs, whereas daily MVPA time demonstrated a negative correlation (*P* < 0.01).

### 3.3 Isotemporal substitution model of SB, LPA, MVPA in different EF RTs groups

After adjusting for total time, isotemporal substitution analysis revealed significant associations between physical activity exchanges and executive function performance (Table 2). Isotemporal replacement of SB by MVPA for 30 min was associated with shorter inhibitory function RTs (B = −0.062, 95%CI: −0.081 to −0.005, *P* < 0.05), working memory RTs (B = −0.787, 95%CI: −1.199 to −0.228, *P* < 0.01), and cognitive flexibility RTs (B = −0.359, 95%CI: −0.471∼ −0.9082, *P* < 0.01). Isotemporal substitution model of LPA by MVPA for 30 min was associated with shorter inhibition RTs (B = −0.023, 95%CI: −0.041 to −0.009, *P* < 0.05), working memory RTs (B = −0.811, 95%CI: −1.411 to −0.298, *P* < 0.01), and cognitive flexibility RTs (B = −0.377, 95%CI: −0.528 to −0.094, *P* < 0.01).

**Table 2:** The isotemporal substitution model of SB, LPA, MVPA on EF RTs.

Subgroups	30 min SB			30 min LPA		
	*B*	*95% CI*	*B*	*95% CI*	*B*	*95% CI*
Inhibitory control RTs						
Substitution- SB	–	–	−0.008	−0.024 to 0.002	−0.062*	−0.081 to −0.005
Substitution- LPA	0.008	−0.002 to 0.024	–	–	−0.023*	−0.041 to −0.009
Substitution- MVPA	0.062*	0.005∼ 0.081	0.023*	0.009 to 0.041	–	–
Working memory RTs						
Substitution- SB	–	–	−0.079*	−0.301 to 0.127	−0.787*	−1.199 to −0.228
Substitution- LPA	0.079*	−0.127 to 0.301	–	–	−0.811**	−1.411 to −0.298
Substitution- MVPA	0.787**	0.228 to 1.199	0.811**	0.298∼ 1.411	–	–
Cognitive flexibility RTs						
Substitution- SB	–	–	−0.051*	−0.153 to 0.078	−0.359**	−0.471 to −0.082
Substitution- LPA	0.051*	−0.078 to 0.153	–	–	−0.377**	−0.528 to −0.094
Substitution- MVPA	0.359**	0.082 to 0.471	0.377**	0.094 to 0.528	–	–

RT, reaction time; SB, sedentary behavior; LPA, low physical activity; MVPA, moderate-to-vigorous physical activity; *P < 0.05, **P < 0.01.

As shown in Table 3, the participants were divided into four groups according to the overall percentile of MVPA time. The linear regression analysis of MVPA on each sub-function of executive function RTs revealed that: In the Q3 (59.02 min/day ≤ MVPA ≤ 60.88 min/day) group, the absolute value of β coefficients of the influence of MVPA on inhibitory function RTs, working memory RTs, and cognitive flexibility RTs was higher than that in Q1 (MVPA < 48.21 min/day), Q2 (48.21 min/day) min/day ≤ MVPA < 59.02 min/day) and Q4 (MVPA >60.88 min/day)groups. The β coefficients were −0.102, −0.147, and −0.128, respectively, and the differences were statistically significant (*P* < 0.01). The MVPA time of 59.02 min/day–60.88 min/day was beneficial to the improvement of the inhibitory control RTs, working memory RTs, and cognitive flexibility RTs.

**Table 3:** The association of MVPA on EF RTs among adolescents.

Subgroups	Q1 (*95% CL*)	Q2 (*95% CL*)	Q3 (*95% CL*)	Q4 (*95% CL*)
Inhibitory control RTs	−0.023 (−0.165 to 0.191)	−0.013 (−0.070 to 0.243)	−0.102 (−0.245 to −0.056)**	−0.016 (−0.157 to 0.069)
Working memory RTs	−0.008 (−2.441 to 3.169)	−0.080 (−1.126 to 5.108)	−0.147 (−5.721 to −1.660)**	−0.011 (−0.448 to 0.312)
Cognitive flexibility RTs	−0.029 (−2.013 to 0.621)	−0.059 (−2.145 to 0.474)	−0.128 (−2.125 to −0.340)**	−0.029 (−0.338 to −0.58)*

RT, reaction time; MVPA, moderate-to-vigorous physical activity; Q1, MVPA < 25th percentile; Q2, MVPA ≥ 25th percentile and <50th percentile; Q3, MVPA ≥ 50th percentile and <75th percentile; Q4, MVPA ≥ 75th percentile; *P < 0.05, **P < 0.01.

**Figure 3:**
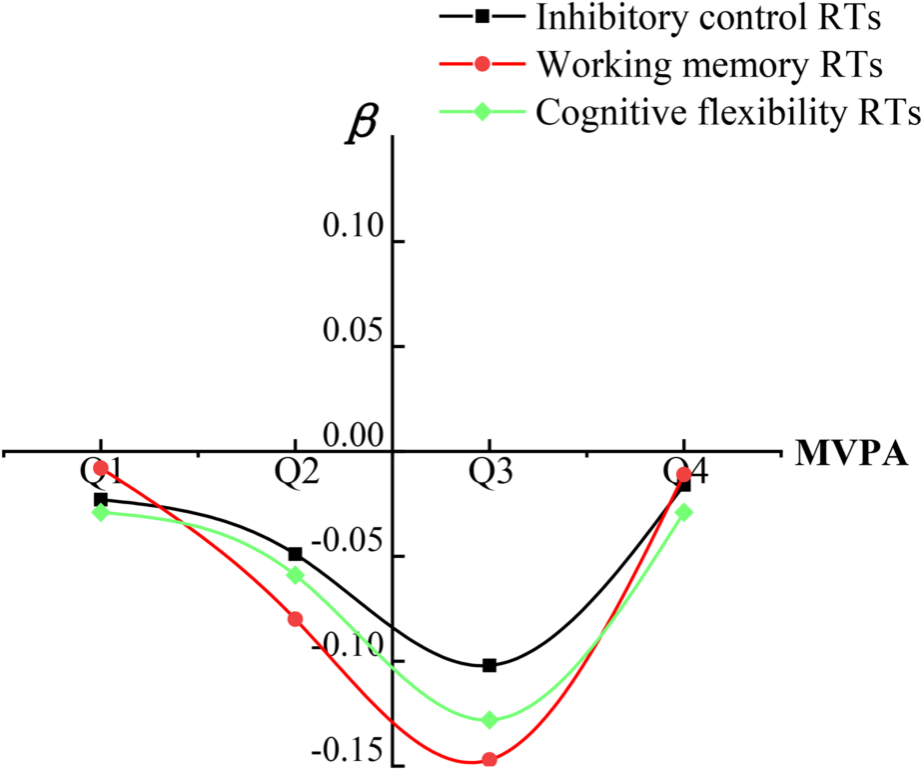
The relationship diagram of MVPA on EF RTs. RT, reaction time; MVPA, moderate-to-vigorous physical activity; Q1, MVPA < 25th percentile; Q2, MVPA ≥ 25th percentile and <50th percentile; Q3, MVPA ≥ 50th percentile and <75th percentile; Q4, MVPA ≥ 75th percentile.

In Figure 3, according to the overall percentile of MVPA time, participants were divided into four groups. The trend analysis confirmed a U-shaped relationship (Figure 3) between MVPA duration and: inhibitory control RTs, working memory RTs, and cognitive flexibility RTs. The optimal MVPA range of 59.02–60.88 min/day showed the most pronounced benefits for all measured executive functions.

## 4 Discussion

Isochronous Substitution Analysis revealed that replacing 30 min of sedentary behavior with 30 min of MVPA significantly reduced reaction times (RTs) in adolescents’ EF tasks (*P* < 0.05). In other words, the study on the relationship of LPA and MVPA on executive function RTs during physical activities has found that MVPA has a more significant promoting association on executive function. This study initially identified an inverted U-shaped relationship between daily MVPA levels and executive function performance. From a neurobiological mechanism perspective, this phenomenon may be attributed to cortical plasticity induced by increased cerebral blood flow, which is modulated by factors such as exercise duration and intensity. Specifically, sustained 60 min MVPA sessions likely optimize executive function enhancement by inducing structural adaptations (e.g., in the prefrontal cortex) through prolonged neural activation. This duration-dependent neuroplastic improvement may explain the observed peak cognitive benefits within this time window (35, 36).

MVPA and TPA showed significant negative associations with inhibitory function RTs (*P* < 0.05), indicating shorter RTs (better performance) with higher activity levels. While, LPA exhibited no significant correlation with inhibitory RTs. Further study found that MVPA showed an inverted “U” -shaped association with the level of inhibitory function RTs of adolescents. Previous studies paid more attention to the relationship between physical activity and inhibitory function RTs of adolescents, while relatively few studies were conducted on the relationship between MVPA and inhibitory function among adolescents. Booth et al. (3) studied the relationship between physical activity and inhibitory function RTs of 4,755 adolescents, showing that compared with adolescents who lacked physical activity, the level of inhibitory function performance of adolescents who have been engaged in medium and high intensity physical activity for a long time was better. A comparative study on physical activity and inhibitory function among Finnish adolescents in Europe found that with the increase of MVPA, the inhibitory function level of adolescents showed a gradual upward trend (37). Ben-Zeev et al. (38) found that MVPA had a significant positive association on inhibitory function in adolescents (*P* < 0.05). In view of previous research analysis, long-term physical activity has a promoting relationship on the inhibitory function of adolescents, and MVPA had a more significant association inhibitory function.

Both MVPA and LPA were associated with improved working memory performance in adolescents, with MVPA demonstrating a stronger dose-response relationship compared to LPA. At present, the research on the correlation between MVPA and working memory among adolescents has been fully confirmed by most scholars. Valken-borghs et al. (39) found that the time increase of MVPA was conducive to the improvement of adolescents’ working memory (*P* < 0.05). A Randomized Controlled Trial (RCT) on working memory in adolescents showed that long-term MVPA in adolescents had a significantly positive impact on working memory performance, and the difference was statistically significant (*P* < 0.05) (38). From the perspective of brain structure, the study analyzed the changing trends of the prefrontal lobe during the completion of working memory tasks across different age groups. The research revealed that the right prefrontal lobe plays a dominant role in 5–6-year-old children during test tasks, while in adults, the left prefrontal lobe is significantly more active during task completion (22). Combined with the results of previous studies and the results of this study, it was found that long-term physical activity had a certain promoting association on the working memory performance of adolescents, and MVPA was more significantly relationship with working memory level than LPA among adolescents.

The results of this study showed that both MVPA and TPA were positively associated with improved cognitive flexibility performance in adolescents. MVPA demonstrated a stronger association with cognitive flexibility enhancement compared to TPA (*P* < 0.05). Early studies mainly focused on the correlation between TPA and cognitive flexibility RTs, but there were few studies on the associations of different physical activity levels on cognitive flexibility RTs. A study showed that the time of MVPA intervention of adolescents for 9 months had a significant improvement association on cognitive flexibility RTs by paired sample t test after intervention. Meanwhile, compared with the independent sample t test, the cognitive flexibility RTs of adolescents in the MVPA intervention group was significantly better than that in the control group, and the difference was statistically significant (*P* < 0.05) (1). Another study on the improvement of MVPA intervention with cognitive flexibility of adolescents in RCT showed that after exercise intervention, the level of cognitive flexibility among adolescents in the intervention group was better than that in the control group, and the difference was statistically significant (*P* < 0.05) (40). Combined with the results of previous studies and the results of this study, long-term regular physical activity was significantly positive on the improvement of adolescents’ cognitive flexibility performance level, and MVPA had a better relationship on the improvement of cognitive flexibility level among adolescents.

### 4.1 Strengths and limitations

On the one hand, many previous studies have investigated the association between PA and EF in adolescents. However, their sample sizes were small and unrepresentative. The strengths of this study include the large sample size (*N* = 4,991) and representative data on the association between PA and EF of adolescents in China. Additionally, we compared the differences between the effects of LPA, MVPA on the EF of adolescents.

On the other hand, this study does have some limitations. As a cross-sectional investigation, it cannot establish a causal relationship between PA and EF, particularly the inability to infer causality.

## 5 Conclusions

Compared with daily LPA time, this study provided evidence that adolescents’ daily MVPA time had a more significant improvement on their inhibitory function, working memory and cognitive flexibility. After controlling for other influencing factors, a “U-shaped” relationship existed between MVPA time and inhibition function RTs, working memory RTs, and cognitive flexibility RTs of adolescents, and an MVPA of 59.02–60.88 min/day had the improvement of the inhibition function, working memory, and cognitive flexibility of adolescents.

## Data Availability

The datasets presented in this study can be found in online repositories. The names of the repository/repositories and accession number(s) can be found in the article/Supplementary Material.

## References

[1] Hillman CH Pontifex MB Castelli DM Khan NA Raine LB Scudder MR Effects of the FITKids randomized controlled trial on executive control and brain function. Pediatrics. (2014) 134(4):e1063–71. 10.1542/peds.2013-3219 25266425 PMC4179093

[2] Booth JN Leary SD Joinson C Ness AR Tomporowski PD Boyle JM Associations between objectively measured physical activity and academic attainment in adolescents from a UK cohort. Br J Sports Med. (2014) 48(3):265–70. 10.1136/bjsports-2013-092334 24149097 PMC3913217

[3] Booth JN Tomporowski PD Boyle JM Ness AR Joinson C Leary SD Associations between executive attention and objectively measured physical activity in adolescence: findings from ALSPAC, a UK cohort. Mental Health Phys Act. (2013) 6(3):212–9. 10.1016/j.mhpa.2013.09.002

[4] Davis CL Tomporowski PD McDowell JE Austin BP Miller PH Yanasak NE Exercise improves executive function and achievement and alters brain activation in overweight children: a randomized, controlled trial. Health Psychol. (2011) 30(1):91–8. 10.1037/a0021766 21299297 PMC3057917

[5] Van Dijk ML De Groot RH Savelberg HH Van Acker F Kirschner PA. The association between objectively measured physical activity and academic achievement in Dutch adolescents: findings from the GOALS study. J Sport Exerc Psychol. (2014) 36(5):460–73. 10.1123/jsep.2014-0014 25356610

[6] Lambourne K Hansen DM Szabo AN Lee J Herrmann SD Donnelly JE. Indirect and direct relations between aerobic fitness, physical activity, and academic achievement in elementary school students. Ment Health Phys Act. (2013) 6(3):165–71. 10.1016/j.mhpa.2013.06.002 25984236 PMC4432844

[7] Hillman CH Erickson KI Kramer AF. Be smart, exercise your heart: exercise effects on brain and cognition. Nat Rev Neurosci. (2008) 9(1):58–65. 10.1038/nrn2298 18094706

[8] Pindus DM Davis RD Hillman CH Bandelow S Hogervorst E Biddle SJ The relationship of moderate-to-vigorous physical activity to cognitive processing in adolescents: findings from the ALSPAC birth cohort. Psychol Res. (2015) 79(5):715–28. 10.1007/s00426-014-0612-2 25351943

[9] Syväoja HJ Kantomaa MT Ahonen T Hakonen H Kankaanpää A Tammelin TH. Physical activity, sedentary behavior, and academic performance in Finnish children. Med Sci Sports Exerc. (2013) 45(11):2098–104. 10.1249/MSS.0b013e318296d7b8 23591292

[10] Hansen DM Herrmann SD Lambourne K Lee J Donnelly JE. Linear/nonlinear relations of activity and fitness with children’s academic achievement. Med Sci Sports Exerc. (2014) 46(12):2279–85. 10.1249/MSS.0000000000000362 24781896 PMC4211996

[11] National Administration of Disease Control and Prevention. People's Republic of China Health Industry Standard: Physical Activity Level Evaluation for Children and Adolescents Aged 7–18 Years. Beijing: National Administration of Disease Control and Prevention (2024). (In Chinese, English abstract]). https://www.cdctj.com.cn/doc/003/001/344/00300134411_cf34f0c2.pdf

[12] Bland JM Altman DG. Measuring agreement in method comparison studies. Stat Methods Med Res. (1999) 8(2):135–60. 10.1177/096228029900800204 10501650

[13] Terwee CB Bot SD de Boer MR van der Windt DA Knol DL Dekker J Quality criteria were proposed for measurement properties of health status questionnaires. J Clin Epidemiol. (2007) 60(1):34–42. 10.1016/j.jclinepi.2006.03.012 17161752

[14] Caspersen CJ Powell KE Christenson GM. Physical activity, exercise, and physical fitness: definitions and distinctions for health-related research. Public Health Rep. (1985) 100(2):126–31. 3920711 PMC1424733

[15] World Health Organization. Global Recommendations on Physical Activity for Health. Geneva: World Health Organization (2010). 26180873

[16] Ainsworth BE Haskell WL Whitt MC Irwin ML Swartz AM Strath SJ Compendium of physical activities: an update of activity codes and MET intensities. Med Sci Sports Exerc. (2000) 32(9 Suppl):S498–504. 10.1097/00005768-200009001-00009 10993420

[17] Chen AG Jiang R Xiaohai JI Tao B Zhu F Yan J. Effects of 8-week moderate fancy rope skipping training on executive function in preadolescent deaf children: a school-based experimental study. Sports & Sci. (2015) 36:105–9. 10.13598/j.issn1004-4590.2015.04.017

[18] Wylie SA Ridderinkhof KR Eckerle MK Manning CA. Inefficient response inhibition in individuals with mild cognitive impairment. Neuropsychologia. (2007) 45(7):1408–19. 10.1016/j.neuropsychologia.2006.11.003 17178419

[19] Smith EE Jonides J. Working memory: a view from neuroimaging. Cogn Psychol. (1997) 33(1):5–42. 10.1006/cogp.1997.0658 9212720

[20] Salthouse TA Atkinson TM Berish DE. Executive functioning as a potential mediator of age-related cognitive decline in normal adults. J Exp Psychol Gen. (2003) 132(4):566–94. 10.1037/0096-3445.132.4.566 14640849

[21] Galmes-Panades AM Varela-Mato V Konieczna J Wärnberg J Martínez-González MÁ Salas-Salvadó J Isotemporal substitution of inactive time with physical activity and time in bed: cross-sectional associations with cardiometabolic health in the PREDIMED-plus study. Int J Behav Nutr Phys Act. (2019) 16(1):137. 10.1186/s12966-019-0892-4 31870449 PMC6929461

[22] Tsujimoto S Yamamoto T Kawaguchi H Koizumi H Sawaguchi T. Prefrontal cortical activation associated with working memory in adults and preschool children: an event-related optical topography study. Cereb Cortex. (2004) 14(7):703–12. 10.1093/cercor/bhh030 15084489

[23] Nieto-López M Sánchez-López M Visier-Alfonso ME Martínez-Vizcaíno V Jiménez-López E Álvarez-Bueno C. Relation between physical fitness and executive function variables in a preschool sample. Pediatr Res. (2020) 88(4):623–8. 10.1038/s41390-020-0791-z 32000261

[24] Freitas LL Cardoso TSG Argollo N. Socioeconomic status, urbanization and executive functions development: differences between urban and rural children. Psicol Teor Pesq. (2022) 38:e38525. 10.1590/0102.3772e38525

[25] Tee J Gan WY Tan KA Chin YS. Obesity and unhealthy lifestyle associated with poor executive function among Malaysian adolescents. PLoS One. (2018) 13(4):e195934. 10.1371/journal.pone.0195934 PMC5903659 29664932

[26] Blair C Kuzawa CW Willoughby MT. The development of executive function in early childhood is inversely related to change in body mass index: evidence for an energetic tradeoff? Dev Sci. (2020) 23(1):e12860. 10.1111/desc.12860 31102547 PMC6859186

[27] Borkertienė V Stasiulis A Zacharienė B Kyguolienė L Bacevičienė R. Association among executive function, physical activity, and weight status in youth. Medicina (Kaunas). (2019) 55(10):677. 10.3390/medicina55100677 31597316 PMC6843179

[28] Costello SE Geiser E Schneider N. Nutrients for executive function development and related brain connectivity in school-aged children. Nutr Rev. (2021) 79(12):1293–306. 10.1093/nutrit/nuaa134 33355357

[29] Malik VS Hu FB. The role of sugar-sweetened beverages in the global epidemics of obesity and chronic diseases. Nat Rev Endocrinol. (2022) 18(4):205–18. 10.1038/s41574-021-00627-6 35064240 PMC8778490

[30] Chinese Research Group on Student Physical Fitness and Health. Association CNSSCH Report on the 2014th National Survey on Students’ Constitution and Health. Beijing: China College & University Press (2016).

[31] Liu Y. Relationship Between BMI, Waist Circumference and Physical Fitness Index of Children and Adolescents. Shanghai: East China Normal University (2019). (In Chinese, English abstract).

[32] Chen YT. Effect of Physical Activity on Executive Function of Children Aged 5–6 Years. Nanjing: Nanjing Normal University (2021). (In Chinese, English abstract).

[33] Quan MH Chen PJ. Dose-effect Relationship between Physical Activity and Healthy Physical Fitness in Preschool Children with Different Cluster Characteristics. Nanjing: The eleventh National Sports Science Congress paper abstracts compilation (2019). (In Chinese, English abstract).

[34] Quan MH Wan CY Zhou T Li LK Chen PJ. Dose-effect relationship between physical activity and physical health in preschool children with different cluster characteristics. Sports Science. (2020) 40(3):7. (In Chinese, English abstract). 10.16469/j.css.202003004

[35] Dyrud JE Donnelly C. Executive function of the ego. Clinical and procedural relevance. Arch Gen Psychiatry. (1969) 20(3):257–61. 10.1001/archpsyc.1969.01740150001001 5764569

[36] García-Molina A. Phineas gage y el enigma del córtex prefrontal [phineas gage and the enigma of the prefrontal cortex]. Neurologia. (2012) 27(6):370–5. 10.1016/j.nrl.2010.07.015 21163195

[37] Syväoja HJ Tammelin TH Ahonen T Kankaanpää A Kantomaa MT. The associations of objectively measured physical activity and sedentary time with cognitive functions in school-aged children. PLoS One. (2014) 9(7):e103559. 10.1371/journal.pone.0103559 25061820 PMC4111611

[38] Ben-Zeev T Hirsh T Weiss I Gornstein M Okun E. The effects of high-intensity functional training (HIFT) on spatial learning, visual pattern separation and attention span in adolescents. Front Behav Neurosci. (2020) 14(14):577390. 10.3389/fnbeh.2020.577390 33093827 PMC7521200

[39] Valkenborghs SR Hillman CH Al-Iedani O Nilsson M Smith JJ Leahy AA Effect of high-intensity interval training on hippocampal metabolism in older adolescents. Psychophysiology. (2022) 59(11):e14090. 10.1111/psyp.14090 35599295 PMC9787522

[40] Wang J. A Comparative Study of the Effects of High Intensity Interval Training and Moderate Intensity Continuous Training on Executive Function and Cardiorespiratory Fitness in 11–12 Year old Adolescents. Shanghai: Shanghai Sport University (2021).

